# Biometeorological conditions at Polish Antarctic Station (King George Island, West Antarctica) according to Universal Thermal Climate Index, 2013–2023

**DOI:** 10.1007/s00484-025-03099-9

**Published:** 2026-02-20

**Authors:** Joanna Plenzler, Katarzyna Piotrowicz, Małgorzata Owczarek

**Affiliations:** 1 Institute of Biochemistry and Biophysics, Department of Antarctic Biology, Polish Academy of Science, Warsaw, Poland; 2 Institute of Geography and Spatial Management, Jagiellonian University, Krakow, Poland; 3 Faculty of Oceanography and Geography, University of Gdansk, Gdansk, Poland

**Keywords:** Universal thermal climate index, UTCI in polar regions, Polar bioclimate, Cold stress, Thermal stress in Antarctica

## Abstract

The Universal Thermal Climate Index (UTCI) was used to estimate thermal stress for humans in the South Shetland Islands (West Antarctica), the region in Antarctica with the highest number of scientific stations and visiting tourists. This was done to analyze the duration of comfortable weather conditions, which are crucial for conducting fieldwork and other outdoor tasks. The UTCI is one of the most commonly used climate thermal indices, but it had not previously been calculated for any region in Antarctica. The research was based on hourly data (air temperature, humidity, global solar radiation and wind speed) derived from the Henryk Arctowski Polish Antarctic Station between 2013 and 2023. The range of hourly UTCI values indicates conditions ranging from no thermal stress to extreme cold stress, with predominant strong cold stress (–26.9 to − 13.0 °C; 40.5% of cases) and very strong cold stress (–39.9 to − 27.0 °C; 25.3% of cases). In such conditions, it is recommended that physical activity is increased and that the extremities and face are protected from the cold while working outside, as well as increasing the thermo-isolation properties of clothing. The same thermal stress class remains for more than one day sporadically. The most favourable conditions for spending time outside (without thermal stress or with slight cold stress), which lasted 7–9 consecutive hours, occurred rarely and only from December to March. Due to high wind speed, some UTCI values fall outside the thermal stress scale; nevertheless, the authors classified these as extreme cold stress and suggest developing the UTCI formula to make it more applicable in regions where very high wind speed occur.

**Supplementary Information:**

The online version contains supplementary material available at 10.1007/s00484-025-03099-9.

## Introduction

**Fig. 1:**
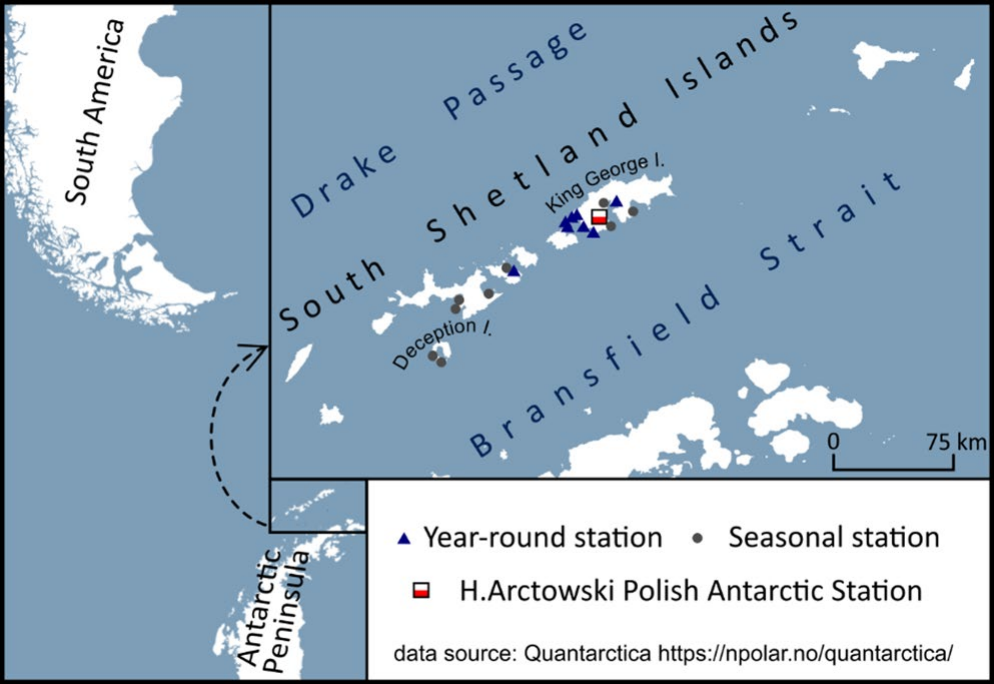
Location of the investigated area

Biometeorological conditions in polar regions are significant in the context of thermal comfort and health protection for workers staying at research stations and vessels, especially during outdoor work. Biometeorological information is also crucial in various aspects of tourism. South Shetland Islands and the northern part of Antarctic Peninsula is the region of Antarctica that is the most visited by cruise ships and yachts (Vessel based operations: Visited sites in Antarctica 2024) and with the highest number of scientific stations (Antarctica Inspected Facilities 2024). It is due to its location relatively close to South American harbors and climate which is not so severe like in the rest of Antarctica. According to Antarctic Treaty Electronic Information Exchange System (2024) from tourist season 2015/2016 to 2022/2023 Deception Island (Fig. 1) was visited by 162 vessels and 23,918 visitors; during the same period Admiralty Bay (King George Island) was visited by 63 vessels and 15,720 visitors. On average it gives almost 3000 visitors on Deception Island and 2000 visitors in Admiralty Bay per tourist season, that is usually from mid-November to February. Tourist activities range from relatively traditional outdoor sports to extreme events such as skiing challenges, skydiving, and scuba diving (Lamers et al. 2007; Snyder 2007). At South Shetland Island there are also 9 year-round and 11 seasonal scientific stations (Antarctica Inspected Facilities 2024; Fig. 1). Assuming at year-round station there are usually 10 person who are hired for the whole year and additional 20 for the summer and that there are ap. 10–20 person at each seasonal station it can be estimated at up to 500 people live at the South Shetland Islands during the summer and ap. 90 during the rest of the year (personal observation).

Planning scientific field works and planning activities for passengers of cruise ships requires good information about possible weather and biometeorological conditions. Biometeorological conditions in Antarctica were usually studied using indices that do not refer directly to physiological responses occurring in the human body under changing thermal conditions of the environment (e.g., Wilson 1967; de Freitas and Symon 1987; Belkin and Karasik 2001; Tyler et al. 2020; Plenzler et al. 2023). Temporally one of the most commonly used biometeorological index is Universal Thermal Climate Index (UTCI, Jendritzky et al. 2012; Potchter et al. 2018; Staiger et al. 2019), which belongs to the bio-thermal indices that consider both meteorological conditions affecting the human body and the associated physiological processes. Generally, there are few studies on thermal comfort using the UTCI index in high latitudes or other regions with cold climate conditions. However, it was applied as a metric of biothermal conditions in the dataset which contains data that covers the whole globe with the grid resolution 0.25°, except the area south from the latitude 60°S. The dataset was computed using meteorological data from the ERA5 reanalysis for the time span that started in January 1940 and is still updated (Di Napoli et al. 2021).

In the Arctic region, the UTCI index was used to explore the inter- and intra-annual changes in thermal stress for people in Alaska and Chukotka (Grigorieva et al. 2023). In another study the UTCI in the two coldest categories (very strong and extreme) was used to identify cold exposure to assess regional aspects of social vulnerability in the context of an extremely cold climatic environment of Alaska (Grigorieva et al. 2024). Data from 456 surface meteorological sites in Alaska, eastern Russia, and northwest Canada for 1979–2017 were used to model hourly UTCI (Mölders 2019). Sikora et al. (2011) appliqued UTCI to assess the perceived climate conditions in the Southwest Svalbard.

Biometeorological conditions at South Shetland Islands were concerned in several research, most of them were conducted with the use of meteorological data from Henryk Arctowski Polish Antarctic Station (thereafter Arctowski Station) and Wind Chill Index (Gregorczuk 1977; Styszyńska 1989, 1994, 2000; Węglowska 2021; Plenzler et al. 2023).

The main goal of this paper is to analyze duration of comfortable weather conditions that are crucial for conducting field works and other outdoor tasks. As a metric of biometeorological conditions we used Universal Thermal Climate Index (UTCI), which was not calculated for the South Shetland Islands or any other place in Antarctica before. Therefore the additional aims of the paper are to supply descriptions of biometeorological conditions in the South Shetland region with UTCI values and to check if those values represent biometeorological conditions in a reliable way.

## Materials and methods

### Research area

**Fig. 2:**
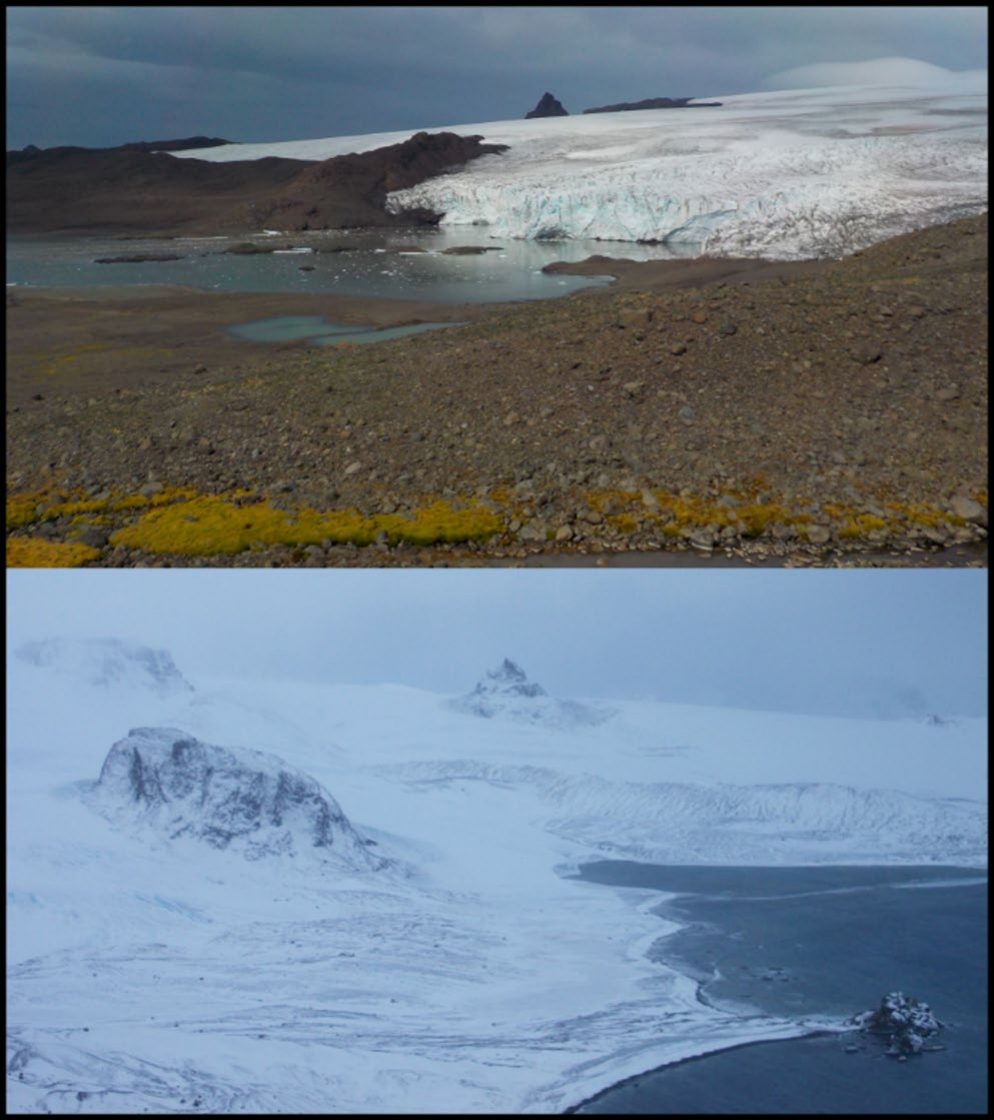
The landscape of King George Island in the vicinity of Henryk Arctowski Polish Antarctic Station in summer (up) and winter (down) photo: J. Plenzler

King George Island is the largest island of South Shetlands and is located approximately 120 km north of the Antarctic Peninsula (Figs. 1 and 2) within the Subantarctic maritime climate zone (Marsz and Styszyńska 2000). The temperature of sea surface, sea ice range and concentration and the lows that often pass through the region are the most important factors that determine climate in this region (Marsz and Styszyńska 2000; Simmonds et al. 2003, Kejna et al. 2013; Turner et al. 2013). Furthermore those factors create meteorological conditions that vary greatly from year to year (Marsz and Styszyńska 2000; Turner et al. 2013).

The region is experiencing some of the most significant climate warming in the world (Kejna 1999; Vaughan et al. 2003; Turner et al. 2005; Stastna 2010; Kejna et al. 2013; Bromwich et al. 2013; Bello et al. 2022; Dalaiden et al. 2022; Casado et al. 2023), which is expected to continue (Fox-Kemper et al. 2021). Consequently, it may be expected that biometeorological conditions will become more favorable for humans in the future. However, considering the complexity of the climate we cannot be certain about the direction of future changes, documentation of the current state of biometeorological condition is important in order to make easier comparisons in the future.

The UTCI is defined as the air temperature of a reference environment that would provide the same physiological response of a reference person as under a given environmental condition specified by the air temperature, wind, humidity, and radiation (Błażejczyk et al. 2010a, b; Jendritzky et al. 2012). The physiological basis of the index is the multi-node model of human body temperature regulation by Fiala (Fiala et al. 2001, 2012; Psikuta et al. 2012; Błażejczyk et al. 2013).

The UTCI values measure the thermal load on the human body and are expressed in degrees Celsius, enabling easy interpretation by a wide range of customers. This index is more complex than the Wind Chill Index, which was used in all previous biometeorological research conducted at Arctowski Station and is calculated only from air temperature and wind speed. As mentioned above, UTCI was calculated for the entire globe, except for the area south of latitude 60° S (Di Napoli et al. 2021). Using this index enables us to compare results with other parts of the world and with previous years, going back as far as 1940.

To objectively assess biometeorological conditions, a thermal stress assessment scale has been constructed (Table 1). Thermal stress is divided into 10 UTCI stress categories ranging from extreme heat stress (above + 46 °C) to extreme cold stress (below − 40 °C). The individual threshold values of the UTCI relate to objective, significant changes in selected physiological parameters of the body, occurring under environmental conditions (Bröde et al. 2012).

**Table 1:** Scale of assessment of thermal stress of the human body according to the UTCI index. Source: Bröde et al. (2012), Błażejczyk et al. (2013)

UTCI [°C] range	Thermal Stress Category (HSC)	Recommendations for protection
> +46.0	4 –EHS Extreme heat stress	Necessary periodical cooling of the organism, necessary supplementation of liquids > 0.5 l per hour; avoid intensive physical activity
+ 38.1 – +46.0	3 – VSHS Very strong heat stress	Necessary periodical use of air-conditioned rooms or shaded areas; necessary supplementation of liquids > 0.5 l per hour; limit physical activity
+ 32.1 – +38.0	2 – SHS Strong heat stress	Necessary supplementation of liquids 0.25 l per hour; recommended use of shaded areas, and periodical limiting of physical activity
+ 26.1 – +32.0	1 – MHS Moderate heat stress	Necessary supplementation of liquids 0.25 l per hour
+ 9.1 – +26.0	0 – TZ No thermal stress	Physiological thermoregulation processes are sufficient to maintain thermal comfort
0.1 – +9.0	−1 – SlCS Slight cold stress	Recommended use of gloves and a hat
˗12.9–0.0	−2 – MCS Moderate cold stress	Intensify physical activity and protect extremities and face from cold
˗26.9– ˗13.0	−3 – SCS Strong cold stress	Intensify physical activity and protect extremities and face from cold; recommended increase in the thermo-isolation properties of clothing
˗39.9 – ˗27.0	−4 – VSCS Very strong cold stress	Intensify physical activity and protect extremities and face from cold; necessary increase in the thermo-isolation properties of clothing, and limited time spent outdoors
≤ ˗40.0	−5 – ECS Extreme cold stress	Limit time spent outdoors to the necessary minimum; necessary increase in the thermoisolation and wind-proof properties of clothing

The verification of the UTCI was conducted in various climatic conditions. In the polar climate, experimental studies were carried out on Spitsbergen, based on which a clear correlation (at the level of 93%) was demonstrated between changes in the temperature of the observer’s cheek skin and changes in thermal conditions determined by the UTCI index (Błażejczyk et al. 2010a, b). Verifications and previous applications of the UTCI indicate that it meets the requirements of a universal index, is valid in various climates and seasons, as well as in spatial and temporal scales, and is useful in a wide range of applications (Jendritzky et al. 2012; Błażejczyk et al. 2013). Attempts were also made to evaluate the sensitivity of the UTCI to meteorological input parameters (Novak 2013, Fröhlich and Matzarakis 2015). A particular sensitivity was demonstrated for wind speed, with the index values quickly decreasing as wind speed increased. The dependency of the UTCI on wind speed seems very strong, especially for lower air temperatures. Due to the method of determining UTCI using the regression formula, recommendations were made to limit the applicability of UTCI for wind speeds above 20 m s^− 1^ and even 17 m s^− 1^. However, it has rightly been emphasized that exposure of the human body surface is minimized because people wear appropriate clothing and do not stay outdoors for long periods. When wind speeds are very high (e.g. 20 m s^− 1^), people tend to stay indoors (Novak 2013). Taking the above comments into account, this study decided to classify very low calculated UTCI values, primarily resulting from high wind speeds, into the category of “extreme cold stress.”

The data used in this research were collected by an automatic weather station located close to Arctowski Polish Antarctic Station situated on the western shore of Admiralty Bay at King George Island (Figs. 1 and 2). Weather station is located on a low sea terrace 2 m a.s.l. and 100 m from the coast. Air temperature, relative air humidity, global solar radiation and wind speed from the period 2013–2023, measured every hour, were used in the research (Table 2).

**Table 2:** Technical characteristics of automatic weather station at Henryk Arctowski Polish Antarctic station

Meteorological element/device	Unit	Manufactured	Type	Accuracy	High [m a.g.l.]
Data logger	-	Campbell	CR3000	-	1.0
Air temperature	°C	Vaisala	HMP155	± 0.2 °C (−40 to −20 °C)	2.0
Relative air humidity	%	Vaisala	HMP155	± 1.3% (−40 to −20 °C)	2.0
Wind speed (2013–2016)	m s^− 1^	Gill Instruments	WindSonic	± 2% at 12 m^− 1^	2.5
Wind speed (2017–2023)					10.0
Solar radiation	W m^− 2^	Kipp&Zonen	CNR4	(360 to 1120 nm) ± 5–10% for daily sums	1.5

The UTCI was calculated using the BioKlima 2.6 software package (Błażejczyk and Błażejczyk 2006) with the implemented computing formula for the mean radiant temperature (°C). Absorbed solar radiation (W m^− 2^) was calculated from the SolGlob submodel (Błażejczyk 2005) depending on total solar radiation (W m^− 2^) and the solar elevation angle (°). The data service of the Astronomical Applications Department of the United States Naval Observatory was used to determine sun elevation at hours during a day (United States Naval Observatory 2024). The software required wind speed measured at the high of 10 m a.g.l. In the case of measurements at a height of 2.5 m (in the period 2013–2016, Table 2), the simplified wind speed reduction formula recommended by the World Meteorological Organization (2021–2023) was used to estimate the wind speed at a height of 10 m. The determined values of the UTCI index were classified taking into account the objective changes in the physiological parameters of the human body, which occur under the influence of atmospheric conditions (Table 1). The times given below refer to Coordinated Universal Time (UTC). Local time at Arctowski Station is UTC − 3 h. In cases where one or more parameters were not recorded by the AWS and were missing from the database, the UTCI was not calculated. The number of hourly UTCI values obtained for each month is shown in Fig. 5. Missing data constitute only 1.8% of the dataset.

## Results

### Meteorological conditions

**Fig. 3:**
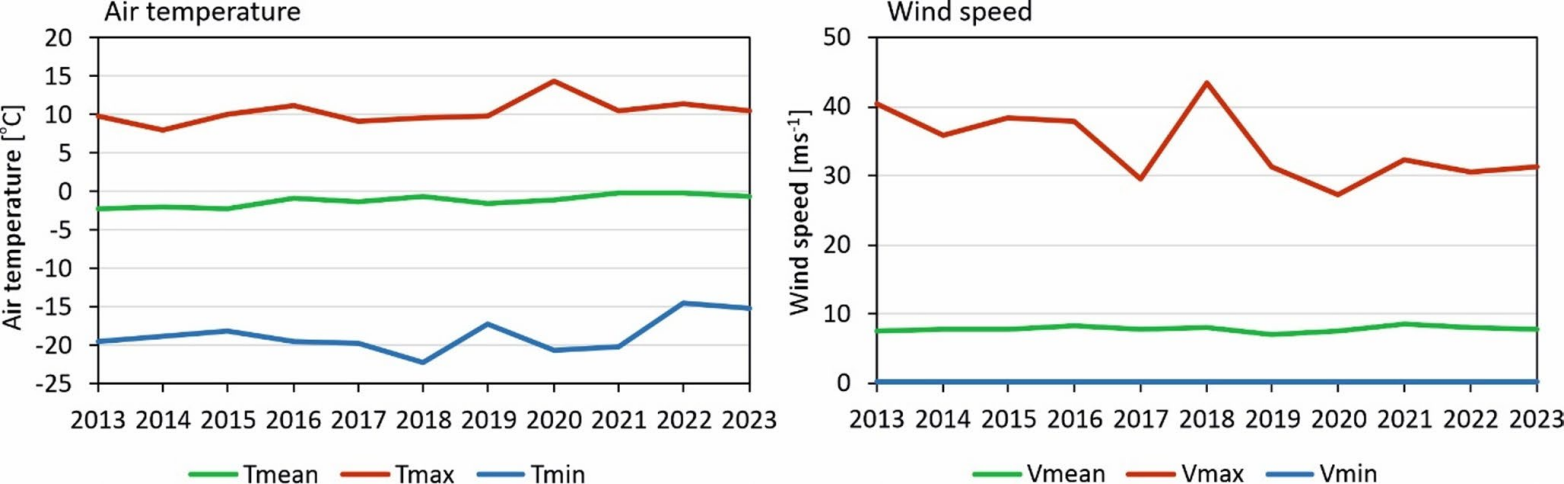
Average annual air temperature and wind speed at 10 m a.g.l. (daily mean, absolute maximum and minimum values) at Arctowski Station (2013–2023)

Prior to the analysis of biometeorological conditions, it is essential to acquire a comprehensive understanding of meteorological conditions per se. According to hourly meteorological data from Arctowski Station from the investigated period (2013–2023) mean annual air temperature there was − 1.2 °C, with the warmest month being February (mean 2.1 °C) and the coldest August (−5.1 °C). For the whole dataset the first quartile was − 3.1 °C, median was − 0.1 °C and the third quartile 1.7 °C. The lowest registered air temperature was − 22.1 °C (in July 2018), and the highest was 14.4 °C (in February 2020). Over the multiannual period, the greatest interannual variability was observed in the minimum temperature (± 0.7 °C), whereas the smallest variability was recorded in the annual mean temperature (2.2 °C) (Fig. 3). Mean annual air humidity was 79.6%, with the actual registered extremes of 17.1% and 98.8%. Mean annual wind speed at 10 m a.g.l. was 7.8 m s^− 1^ (2013–2023), and a maximum registered at that period was 43.5 m s^− 1^ (Fig. 3). Furthermore, wind speeds exceeding 5 ms^− 1^ occurred for 63.1% of the total hours. In 23% of cases, the velocity exceeded 10 m s^− 1^. At the Arctowski Station, the longest day lasts 19 h and 50 min, with a solar noon altitude of 51.3° on 21 December. In contrast, the shortest day lasts 5 h and 5 min, with a solar noon altitude of 4.6° on 20 June (United States Naval Observatory 2024). According to measurements taken at 10-minute intervals from 2018 to 2023, the mean daily sum of solar radiation is 18.98 MJ m^− 2^ in December and 0.38 MJ m^− 2^ in June (Plenzler et al. 2024).

## Annual course of UTCI values

**Fig. 4:**
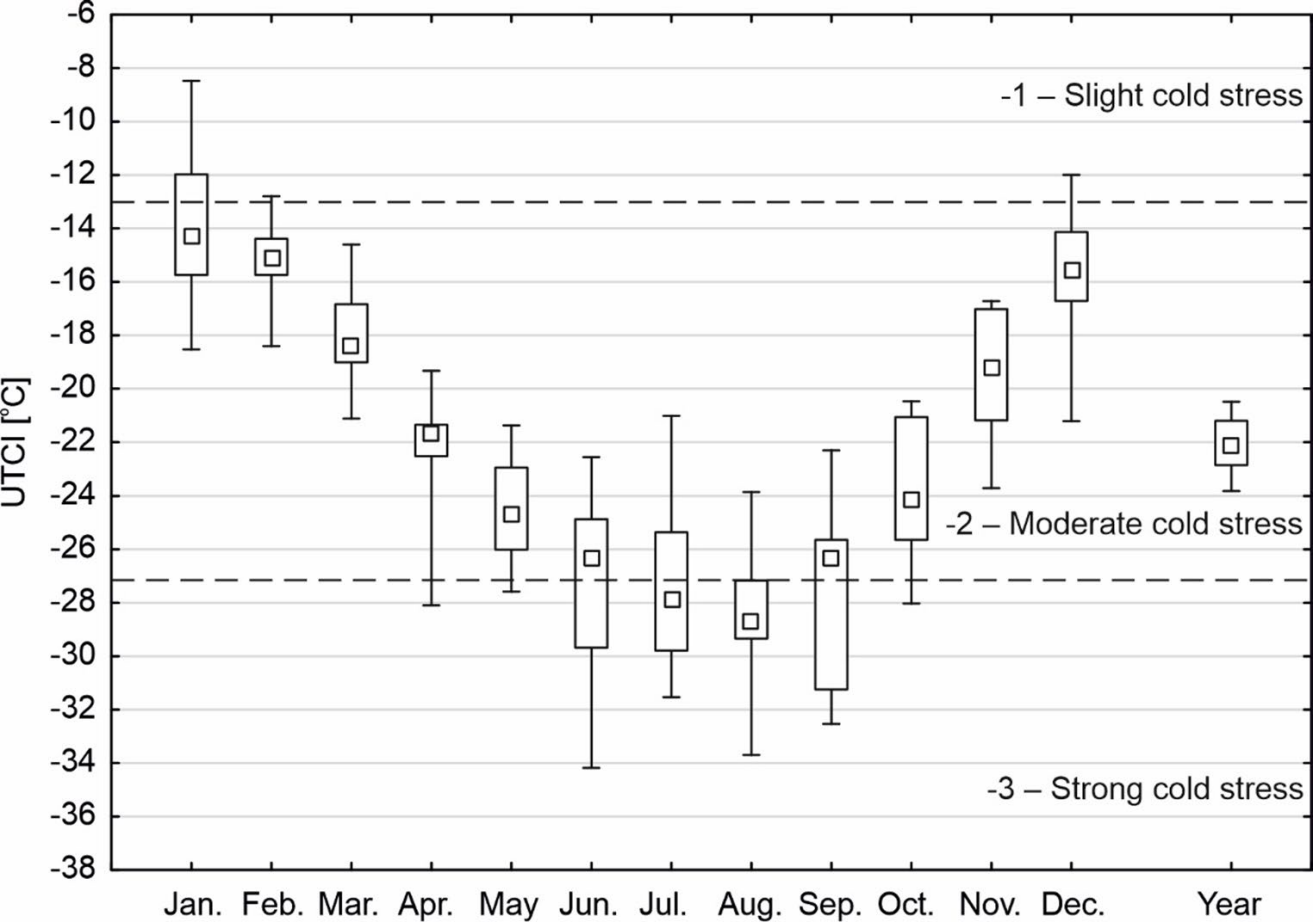
Range of variability of mean monthly UTCI values at Arctowski Station (2013–2023). The dashed line shows the limits of scale of assessment of thermal stress

During 2013–2023 the mean annual and monthly UTCI at the Arctowski Station reached values from − 34.2 °C (June 2018) to −8.2 °C (January 2020; Fig. 4). Most of the mean monthly values corresponded to the moderate cold stress category (−2), while from April to October they also correspond to the strong cold stress category (−3) (Table 1; Fig. 4). Mean monthly UTCI values were most variable (more than 10 °C) for two winter months (June and July) and for September and January. The least variable (7 °C) monthly mean UTCI values were recorded in February, March and May (Fig. 4).

**Fig. 5:**
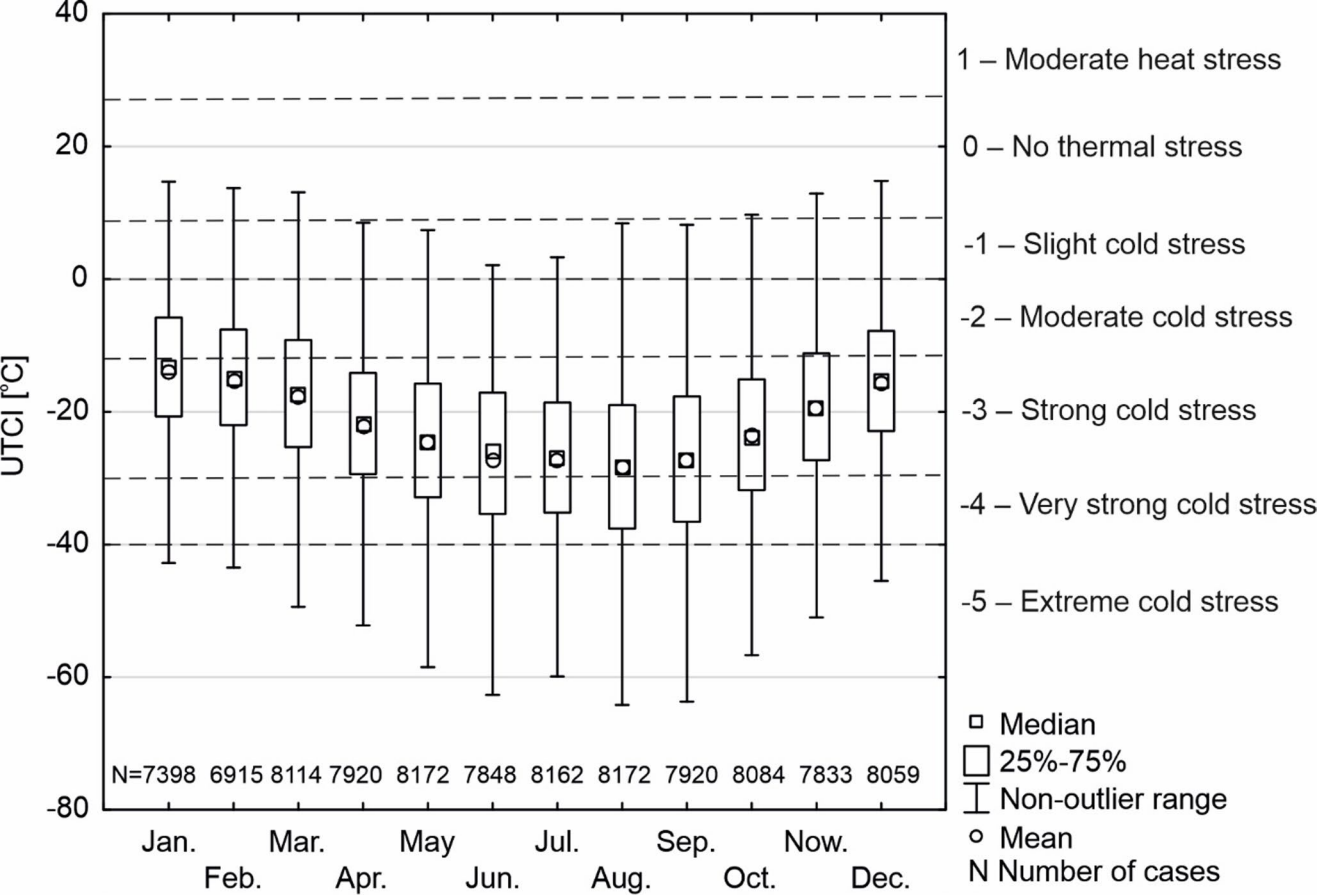
Range of variability of actual hourly UTCI values at Arctowski Station (2013–2023). The dashed line shows the limits of scale of assessment of thermal stress

Hourly UTCI values across individual months most frequently (25th − 75th percentile) fell within two thermal stress categories: moderate to strong cold stress (November-March) and strong to very strong cold stress (April-October) (Fig. 5). Among extreme and outlier values, 8.2% of hourly UTCI values recorded between 2013 and 2023 were below − 40.0 °C, thus classified as extreme cold stress (Table 1). Of these, 80.5% ranged between − 40.0 °C and − 50.0 °C, 19% between − 50.1 °C and − 100.0 °C, and in 0.6% of cases (44 h), UTCI dropped below − 100.1 °C. In all of these extreme cases, wind speed exceeded 31 m·s⁻¹, which largely accounted for the extremely low UTCI values. The most extreme value (−3261.9 °C) was recorded on 30 June 2018 at 20:00 UTC, when the air temperature was − 10.2 °C and the wind speed reached 43.5 m s⁻¹. Despite prior recommendations limiting UTCI calculations to conditions where wind speed does not exceed 20 m s⁻¹, we chose to include these values in the extreme cold stress category.

## Daily course of thermal stress according to UTCI

**Fig. 6:**
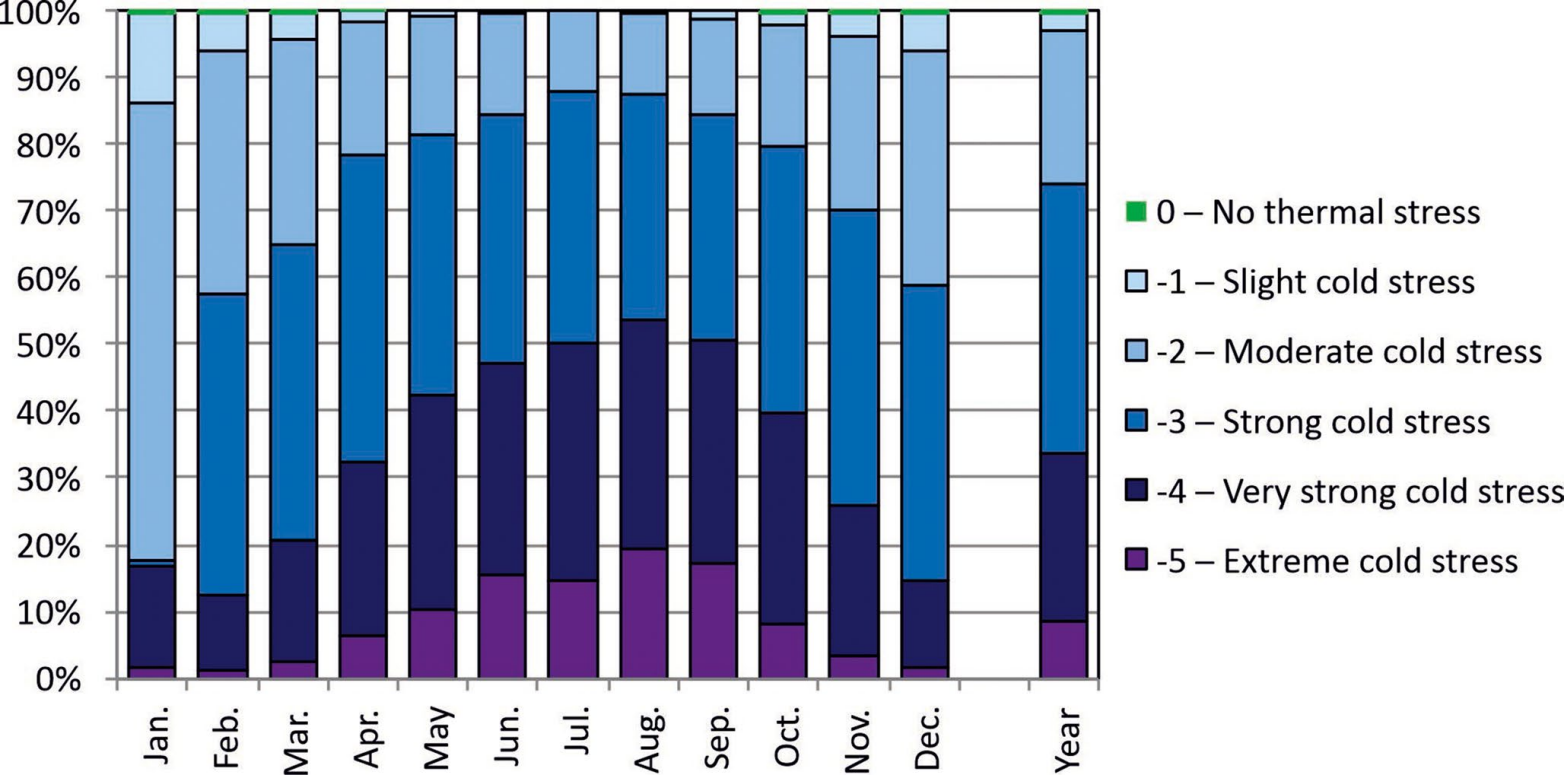
Frequency of occurrence (%) of thermal stress categories according to UTCI hourly values at Arctowski Station (2013–2023)

The range of hourly UTCI values during the analyzed period indicates thermal stress conditions from no thermal stress (category 0) to extreme cold stress (−5) (Figs. 4 and 5). The predominant thermal stress category was strong cold stress (−3), accounting for 40.5% of all cases annually (ranging from 33.9% in August to 46.0% in April) (Fig. 6). Very strong cold stress and extreme cold stress (categories − 4 and − 5) together comprised 30.8% of the cases, most frequently occurring during the winter months (July-August, approximately 50%). The strong cold stress category was dominant (34–46% of the cases) from February to July and from September to December. In January the most frequent category was moderate cold stress (−2; 41.0%), while in August – very strong cold stress (−4), which constituted 34.3% of cases (Fig. 6).

Thermoneutral conditions, the mildest that occurred at the Arctowski station during 2013–2023, occurred infrequently, in only 0.3% of the hours during the period from October to April. (Fig. 6). These individual cases were registered from 9:00 to 22:00, but most frequently from 15:00 to 18:00 (Tab. S1a). Extreme cold stress conditions (−5) occurred with significantly higher frequency - potentially they can occur at almost any time of the month. During the study period, such conditions did not occur only in February at 2:00 (Tab. S1b), but most frequently in August at 11:00 and 12:00.

**Fig. 7:**
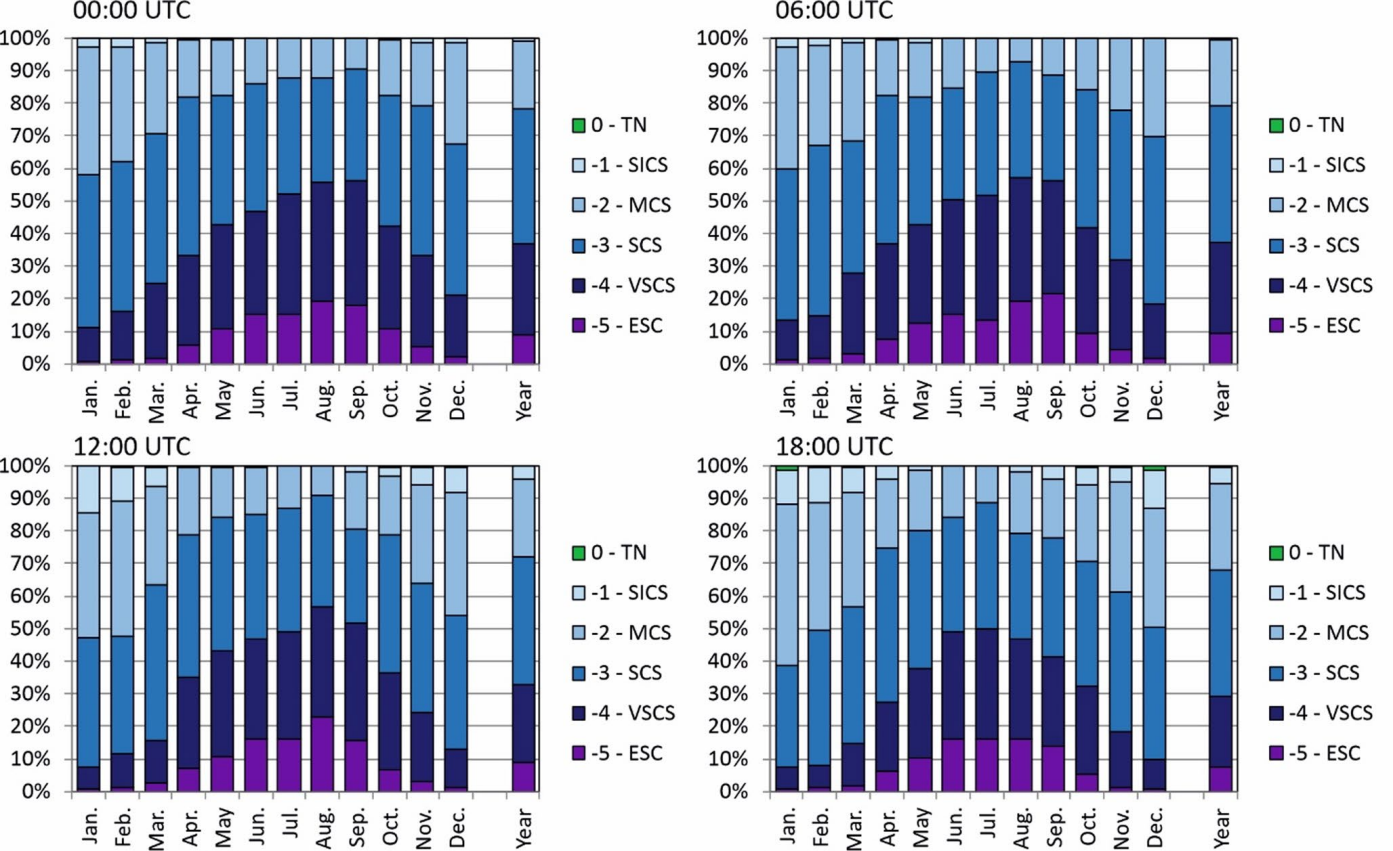
Frequency of occurrence (%) of various thermal stress categories according to UTCI in particular hours at Arctowski Station (2013–2023)

To better understand biometeorological conditions in the vicinity of Arctowski Station attention was paid to the frequency of particular thermal stress categories – and it was analyzed at three-hour intervals, representing different parts of the day (Fig. 7, Tab. S2). In the summer half-year (October to March) between 9:00–18:00 moderate and strong cold stress (−2 and − 3) was registered for more than 50% of hours (Tab. S2). The thermoneutral conditions, that are favorable for working outside, occurred most frequently from October-November to February-March between 9:00–18:00 (6:00–15:00, and sporadically to 21:00 (Tab. S2). However it should be noted that at the same months and time very strong cold stress (−4), may occur with the frequency of 5%, and extreme cold stress (−5) with the frequency of 1%.

In the winter half year (April-September) the cold stress (−3) and very cold stress (−4) categories were dominant, together they made up more than 60% of cases. The extreme cold stress (−5) appeared even during 9:00–15:00, and it made up 10–20% of cases. In that half year the slight cold stress (−1) may appear only between 12:00–18:00 with a frequency of 1–5% (Fig. 7, Tab. S2).

### Stability of the thermal stress conditions

The final stage of the study of thermal stress to which the human body may be exposed when staying in the vicinity of the Arctowski Station was to analyze sequences of hours in which the categories were the same, i.e. belonged to values within a single range. A distinction was made between cases in which a particular class of thermal stress occurred at one hour only, followed by cases at 2–6, 7–12 and 13–24 h, and cases at 25–48 h (2 days) and 49–72 h (3 days) (Table 3). Both in the year and in the individual half-years, thermoneutral conditions (0) most often occurred in single hours. Only occasionally did they form a sequence of up to 6 h (Table 3). Slight cold stress (−1), conditions that occurred at a frequency of only 2.7% of all cases in the year, usually last only for one hour (74% of category cases). Sequences of that category that lasted up to 6 h occurred more often in summer half-year; sequences up to 12 h occurred very rarely and only in summer-half year (0,8%, Table 3).

**Table 3:** Frequency of occurrence (%) of sequences of body thermal stress classes in different length intervals (in hours) at Arctowski station (2013–2023)

January-December										
Hours/Categories	0		−1		−2		−3	−4		−5
1	96.1		74.1		52.6		44.4	49.3		56.3
2–6	3.9		25.1		41.5		48.6	44.6		34.9
7–12	-		0.8		4.7		6.0	5.3		5.2
13–24	-		-		1.0		0.9	0.9		2.4
25–48	-		-		0.1		0.1	0.0		1.0
49–72	-		-		-		-	-		0.2
sum	100.0		100.0		100.0		100.0	100.0		100.0
April-September										
Hours/Categories	0		−1		−2		−3	−4		−5
1	100.0		81.8		59.0		45.5	47.3		53.8
2–6	-		18.2		37.0		48.7	46.5		36.0
7–12	-		-		3.0		5.2	5.4		6.0
13–24	-		-		0.9		0.6	0.8		2.9
25–48	-		-		0.1		0.1	0.0		1.1
49–72	-		-		-		-	-		0.3
sum	100.0		100.0		100.0		100.0	100.0		100.0
October-March										
Hours/Categories	0		−1		−2		−3	−4		−5
1	99.1		51.4		12.5		9.2	20.2		64.6
2–6	0.9		47.0		75.6		77.8	69.4		31.2
7–12	-		1.6		9.9		11.1	8.5		2.6
13–24	-		-		2.0		1.8	2.0		0.8
25–48	-		-		0.1		0.1	-		0.8
49–72	-		-		-		-	-		-
sum	100.0		100.0		100.0		100.0	100.0		100.0

Moderate cold stress conditions (−2) similar to very strong cold stress conditions appeared with frequency of 25% during the year. In summer half year both those categories usually create sequences up to 6 h, but during winter half year they usually appear only for one hour (Table 3). The most common thermal stress in the vicinity of Arctowski Station - strong cold stress (−3), also clustered in a 2–6 h sequences, especially during summer half year when such sequences constituted almost 78% cases from that category (Table 3). In terms of the duration of the thermal stress category, it was the class with extreme cold stress (−5) that occurred the longest, i.e. among the others, it created the most stable biometeorological conditions during certain periods of the year. Such conditions clustered into sequences lasting up to 68 h. Such long-lasting (2–3 days) and simultaneously extremely stressful cases occurred in the winter half of the year with a frequency of 0.3% (Table 3).

For the benefit of the personnel of scientific stations and vessels and visitors to the area, the bioclimatic conditions were analyzed in greater detail to ascertain the continuity of conditions with minimal stress on the body (0 and − 1). Such conditions permit outdoor activity with minimal risk of frostbite. The analyses show that these most favorable biometeorological conditions (0 and − 1) occur most frequently alone (75.5% of the cases), while they last the longest (9 h) (Table 4). There is a clear increase in their frequency throughout the year from November to March (Table 4).

**Table 4:** Number of single hours and their sequences with duration from 2 to 9 h in individual months without thermal loads (with thermoneutral conditions – 0) or slight cold stress (−1) at Arctowski station (2013–2023)

Length of sequence hours	Jan.	Feb.	Mar.	Apr.	May	Jun.	Jul.	Aug.	Sep.	Oct.	Nov.	Dec.	Year	%
1	278	217	183	86	44	6	5	21	52	91	179	214	1376	75.4
2–6	106	65	58	18	5	1	-	4	19	29	50	82	437	23.9
7–9	5	3	1	-	-	-	-	-	-	-	-	4	13	0.7
sum	389	285	242	104	49	7	5	25	71	120	229	300	1826	100.0

A little more practical information about biothermal conditions can be obtained by comparing the frequency of conditions with little or no cold stress over the course of the day (Tab. S3). On average over the year, the conditions most conducive to outdoor activity occurred between 11.00 and 19.00 UTC, especially in December and January. In these months, the frequency of longer sequences, 2 to 6 h long, reached about 2% (Tab. S3). Considering the most stable conditions without thermal stress or with slight cold stress (0 or −1), lasting 7–9 consecutive hours, their occurrence is limited to only four months (Dec.-Mar.). The onset of such sequences of hours with stable conditions occurred most frequently between 10.00 and 16.00 UTC. In the analyzed multiannual period, such sequences of hours with favorable conditions also occurred at night. From 19.00 to 1.00 UTC, for the next 8–9 h, the biometeorological conditions were stable and favorable for outdoor activities. However, such situations occurred only in January and not in every year.

## Discussion

The UTCI can be used to investigate a variety of research questions, one of which is the threat to health. In cold conditions, the most important factors are the body’s temperature, especially of the hands and face, and the process of shivering thermogenesis. For example: under conditions corresponding to a UTCI value of 9 °C, there is a decrease in hand temperature requiring gloves, at a UTCI of 0 °C, the rate of skin blood flow decreases, and at −10 °C, a drop in internal temperature begins. Among the major consequences of cold stress, tissue injury resulting from tissue cooling and subsequent freezing (frostbite) and hypothermia are the most common. Other impacts of cold stress include an increase in respiratory, cardiovascular, and circulatory risks, as well as adverse effects on concentration and memory (Holmer et al. 2012, Skutecki et al. 2019). At Arctowski Station almost all of the registered hourly cases had UTCI below 9 °C (Tab. S1a), which means that almost all the time biothermal conditions may cause health threats to the person that is not properly dressed. This is consistent with conclusions obtained with the application of Wind Chill Index (Plenzler et al. 2023). However it should be kept in mind that it is doubtful to compare conditions described by two different indices, that are built on different conceptual assumptions. The WCI is one of the simplest biometeorological indices, it was designed to assess the cooling effect of wind at low air temperatures. It is intended primarily to account for convective heat losses. It considers only two variables: air temperature and wind speed (usually reduced to the level of an adult torso, i.e. approximately 1.2 m) and is calculated using a simple mathematical formula. Consequently, each index value corresponds to the thermal sensation of a person wearing clothing with an insulation value of 4 clo. By contrast, the UTCI is derived from a multi-node model that incorporates all major meteorological factors and modes of energy transfer, as well as physiological processes. It enables evaluation of the actual thermal load on the human body, not just the subjective thermal sensation, and it has a much broader range of applicability across diverse weather conditions. Nevertheless they gave a similar general overview, only conditions described by UTCI seems to be a little bit more severe, which is reasonable since this index takes into account more factors and is more complex.

Most of the work carried out by scientific station staff requires them to work outside, either in the field or maintaining the station’s buildings and equipment. For fieldwork in the immediate vicinity of the station that doesn’t require the use of sea transport, 6 h is usually sufficient to complete the tasks. For fieldwork that requires the use of sea transport (motorboat) or travel across the glacier, 6 h is the shortest possible time. Nevertheless, considering the volatility of nature in polar regions, it is good to have a margin of up to 12 h (personal observation). As shown in the results section, for each cold stress category, a significant majority of sequences last up to 6 h. Moreover, conditions with minimal stress on the body (classes 0 and − 1) occurred with a very low frequency and only from December to March, most frequently in the middle of the day in December and January. This confirms the high variability of biothermal conditions that were described in other research (Plenzler et al. 2023) and general observations of personnel of the Arctowski Station. In the light of the present duration of thermal stress time sequences a person that works or travels in the vicinity of Arctowski Station must be prepared that the biometeorological sensations will change during the stay outside.

Compared to the research conducted at Svalbard (Sikora et al. 2011), conditions at King George Island seem to be more severe. In that research the value of the UTCI ranged from − 7.2 °C (in January) to 16.5 °C (in June). The frequency of occurrence of thermal stress categories varied depending on the month and location of measurement points with different local climate factors. From September to May, strong or very strong cold stress conditions were most frequent, with a frequency of 30–94%. The category of no thermal stress appeared from May to September, most often on the firn field (8%), where the intensity of the solar radiation energy flux is significant for the amount of this form of energy absorbed by the human body. In general, same as Arctowski Station, strong and very strong thermal stress conditions also appeared the most often there, but during Svalbard summer “no thermal stress” conditions occurred more often than during relevant time at King George Island. Since Svalbard is located at a higher latitude than King George Island one of the reasons for that is the intensity of solar radiation during summer months.

Most often the UTCI thermal stress categories obtained for Arctowski Station were similar as in the presented research conducted in Alaska, Chukotka and northern Canada but the important difference is that in comparison with those regions, at King George Island category no thermal stress occurred very seldom (Tab. S1, Grigorieva et al. 2023; Mölders 2019).

It should be kept in mind that our results are limited to a small area, since they are based on data from only one meteorological station. However, there is a correlation between the daily values of the meteorological parameters at the meteorological stations on King George Island (Plenzler et al. 2024) and the South Shetland Islands (Bañón et al. 2013). The hourly course may not be consistent due to local factors that influence the meteorological conditions, such as the location above sea level and the amount of solar radiation, which is limited by the surrounding terrain. Further investigation of biometeorological conditions in the area, based on data from a larger area, is needed.

Another limitation of the presented research is that the data series is quite short, which does not allow for the study of the variability of biothermal conditions over several years. According to Grigorieva et al. (2023), global warming positively impacts the climatology of thermal stress in the Arctic, providing advantages for the development of tourism and recreation. In future, it would be interesting to investigate this topic using longer time series than those used in this research. In the context of current climate warming, analyzing extreme values, both high and low, could also be an interesting topic. Furthermore, we suggest that future studies of human biometeorology in the South Shetland Islands should consider the thermal sensations experienced by people working there.

## Conclusions

In the paper we presented mean monthly and actual hourly values of UTCI at Henryk Arctowski Polish Antarctic Station, located at King George Island (West Antarctica). The predominant thermal stress category there was strong cold stress sensation (UTCI from − 26,9 °C to −12.9 °C) followed by very strong cold stress sensation (UTCI from–39.9 to − 27.0 °C). UTCI values for those categories are significantly lower than air temperature in that area. In the vicinity of Arctowski Station biometeorological conditions defined by UTCI vary a lot. Seldom the weather is stable through the whole day, moreover sporadically thermal stress class remains the same for 2 or 3 days. The mildest conditions – namely thermoneutral appear only for separate hours or last only up to 6 h. In such circumstances it is imperative that meticulous planning is undertaken for any outdoor activities that may be conducted by scientific stations and vessel personnel, in addition to tourists visiting the area. Moreover the individuals partaking in outdoor activity are attired in appropriate winter clothes.

Considering the quite high occurrence of extremely low UTCI values that were determined by wind speed that exceeded 20 m s^− 1^, we propose that consideration be given to the inclusion of an additional category of cold stress that may be relevant to polar regions. That may be of use in the assessment of biothermal conditions in locations where there is no permanent human habitation, but where human populations are present only during certain periods of the year (e.g. scientific stations) or only on sporadic occasions (e.g. scientific or adventure expeditions).

The application of UTCI, a method now widely employed to estimate biometeorological conditions, facilitates the comparison of biometeorological conditions at South Shetland Islands with those observed in other regions worldwide. This may be useful in education, especially when comparing with permanently inhabited areas.

## Supplementary Information

Below is the link to the electronic supplementary material.

ESM1 (DOCX 53.7 KB)

## Data Availability

The data are available upon request, which can be made by contacting the corresponding author.
