# Supplementary material for: Biometeorological conditions at Polish Antarctic Station (King George Island, West Antarctica) according to Universal Thermal Climate Index, 2013–2023
Source: Int J Biometeorol. 2026 Feb 20;70(3):69. doi: 10.1007/s00484-025-03099-9 (PMC12923444; doi:10.1007/s00484-025-03099-9)
Supplement: Supplementary file 1 — (DOCX 53.7 KB) [file 484_2025_3099_MOESM1_ESM.docx]

**Supplementary material**

**Table S1** Number of cases without thermal stress (a) and with extreme cold stress (b) per day in individual months at Arctowski Station (2013-2023)

a) No thermal stress (Thermoneutral zone)

| Month | Hours (UTC) | | | | | | | | | | | | | | | | | | | | | | | | Sum |
| --- | --- | --- | --- | --- | --- | --- | --- | --- | --- | --- | --- | --- | --- | --- | --- | --- | --- | --- | --- | --- | --- | --- | --- | --- | --- |
|  | 0 | 1 | 2 | 3 | 4 | 5 | 6 | 7 | 8 | 9 | 10 | 11 | 12 | 13 | 14 | 15 | 16 | 17 | 18 | 19 | 20 | 21 | 22 | 23 |  |
| Jan. | - | - | - | - | - | - | - | - | - | 2 | - | - | - | - | 1 | 2 | 4 | - | 4 | 1 | 4 | - | 1 | - | 19 |
| Feb. | - | - | - | - | - | - | - | - | - | 1 | - | - | 2 | 1 | 2 | 4 | 1 | 6 | 2 | - | 3 | 2 | - | - | 24 |
| Mar. | - | - | - | - | - | - | - | - | - | - | - | - | 1 | 1 | - | - | 1 | 1 | 2 | 1 | - | - | - | - | 7 |
| Apr. | - | - | - | - | - | - | - | - | - | - | - | - | - | - | 1 | - | - | 1 | - | 1 | - | - | - | - | 3 |
| May | - | - | - | - | - | - | - | - | - | - | - | - | - | - | - | - | - | - | - | - | - | - | - | - | - |
| Jun. | - | - | - | - | - | - | - | - | - | - | - | - | - | - | - | - | - | - | - | - | - | - | - | - | - |
| Jul. | - | - | - | - | - | - | - | - | - | - | - | - | - | - | - | - | - | - | - | - | - | - | - | - | - |
| Aug. | - | - | - | - | - | - | - | - | - | - | - | - | - | - | - | - | - | - | - | - | - | - | - | - | - |
| Sep. | - | - | - | - | - | - | - | - | - | - | - | - | - | - | - | - | - | - | - | - | - | - | - | - | - |
| Oct. | - | - | - | - | - | - | - | - | - | - | - | - | 1 | - | - | 3 | - | 4 | 1 | - | - | - | - | - | 9 |
| Nov. | - | - | - | - | - | - | - | - | - | 1 | - | - | 2 | 4 | 2 | 3 | 4 | 1 | 1 | 2 | - | - | - | - | 20 |
| Dec. | - | - | - | - | - | - | - | - | - | - | 1 | 2 | 2 | 2 |  | 2 | 5 | 4 | 4 | 3 | 1 | - | - | - | 26 |
| Year | - | - | - | - | - | - | - | - | - | 4 | 1 | - | 8 | 8 | 6 | 14 | 15 | 17 | 14 | 8 | 8 | 2 | 1 | - | 108 |

b) Extreme cold stress

| Month | Hours (UTC) | | | | | | | | | | | | | | | | | | | | | | | | Sum |
| --- | --- | --- | --- | --- | --- | --- | --- | --- | --- | --- | --- | --- | --- | --- | --- | --- | --- | --- | --- | --- | --- | --- | --- | --- | --- |
|  | 0 | 1 | 2 | 3 | 4 | 5 | 6 | 7 | 8 | 9 | 10 | 11 | 12 | 13 | 14 | 15 | 16 | 17 | 18 | 19 | 20 | 21 | 22 | 23 |  |
| Jan. | 2 | 4 | 2 | 2 | 6 | 3 | 4 | 2 | 3 | 3 | 2 | 2 | 3 | 2 | 3 | 1 | 1 | 1 | 3 | 2 | 3 | 3 | 2 | 4 | 63 |
| Feb. | 3 | 4 | - | 3 | 5 | 4 | 5 | 5 | 4 | 3 | 7 | 2 | 3 | 3 | 3 | 2 | 3 | 2 | 3 | 2 | 2 | 4 | 1 | 1 | 74 |
| Mar. | 6 | 10 | 10 | 6 | 9 | 11 | 10 | 12 | 7 | 8 | 7 | 6 | 9 | 7 | 7 | 8 | 8 | 6 | 6 | 6 | 8 | 11 | 14 | 6 | 198 |
| Apr. | 19 | 19 | 18 | 19 | 23 | 17 | 25 | 22 | 26 | 23 | 21 | 20 | 23 | 22 | 22 | 18 | 16 | 19 | 20 | 20 | 25 | 21 | 26 | 25 | 509 |
| May | 37 | 35 | 35 | 35 | 37 | 37 | 43 | 34 | 35 | 33 | 33 | 38 | 36 | 38 | 35 | 31 | 30 | 28 | 35 | 36 | 41 | 37 | 36 | 35 | 850 |
| Jun. | 50 | 52 | 53 | 48 | 47 | 47 | 50 | 43 | 48 | 60 | 52 | 52 | 53 | 53 | 52 | 46 | 50 | 50 | 53 | 47 | 43 | 48 | 52 | 48 | 1197 |
| Jul. | 52 | 47 | 42 | 43 | 41 | 48 | 46 | 53 | 49 | 51 | 48 | 54 | 55 | 60 | 50 | 49 | 39 | 42 | 54 | 55 | 54 | 52 | 52 | 41 | 1177 |
| Aug. | 66 | 65 | 68 | 68 | 72 | 73 | 66 | 70 | 77 | 74 | 64 | 82 | 78 | 65 | 61 | 56 | 49 | 53 | 55 | 62 | 71 | 69 | 70 | 65 | 1599 |
| Sep. | 59 | 61 | 52 | 53 | 61 | 66 | 71 | 59 | 66 | 65 | 62 | 62 | 52 | 50 | 46 | 43 | 41 | 36 | 45 | 50 | 61 | 65 | 63 | 59 | 1348 |
| Oct. | 36 | 36 | 35 | 31 | 30 | 31 | 31 | 25 | 34 | 30 | 33 | 30 | 23 | 20 | 20 | 20 | 23 | 21 | 17 | 23 | 23 | 25 | 27 | 39 | 663 |
| Nov. | 17 | 17 | 17 | 7 | 15 | 17 | 14 | 19 | 13 | 13 | 11 | 11 | 10 | 7 | 6 | 6 | 6 | 5 | 4 | 7 | 5 | 7 | 7 | 12 | 253 |
| Dec. | 7 | 7 | 7 | 10 | 7 | 7 | 5 | 6 | 5 | 3 | 3 | 5 | 4 | 4 | 2 | 1 | 1 | 2 | 3 | 4 | 3 | 5 | 8 | 11 | 120 |
| Year | 354 | 357 | 339 | 325 | 353 | 361 | 370 | 350 | 367 | 366 | 343 | 364 | 349 | 331 | 307 | 281 | 267 | 265 | 298 | 314 | 339 | 347 | 358 | 346 | 8051 |

**Table S2** Frequency (%) of various thermal stress categories according to UTCI in particular hours at Arctowski Station (2013-2023)

| 00:00 UTC | | | | | | |  | 03:00 UTC | | | | | | |  | 06:00 UTC | | | | | | | | | |
| --- | --- | --- | --- | --- | --- | --- | --- | --- | --- | --- | --- | --- | --- | --- | --- | --- | --- | --- | --- | --- | --- | --- | --- | --- | --- |
| Month | Thermal stress categories | | | | | |  | Month | Thermal stress categories | | | | | |  | Month | | Thermal stress categories | | | | | | | |
|  | 0 | -1 | -2 | -3 | -4 | -5 |  |  | 0 | -1 | -2 | -3 | -4 | -5 |  |  |  | 0 | -1 | -2 | | -3 | -4 | -5 | |
| Jan. | - | 2.6 | 39.3 | 46.8 | 10.7 | 0.6 |  | Jan. | - | 2.9 | 33.4 | 48.4 | 14.6 | 0.6 |  | Jan. | | - | 2.6 | 37.3 | | 46.8 | 12.0 | 1.3 | |
| Feb. | - | 2.8 | 35.1 | 46.2 | 14.9 | 1.0 |  | Feb. | - | 1.0 | 35.1 | 50.7 | 12.2 | 1.0 |  | Feb. | | - | 2.1 | 30.9 | | 52.1 | 13.2 | 1.7 | |
| Mar. | - | 1.5 | 27.9 | 46.0 | 22.9 | 1.8 |  | Mar. | - | 1.8 | 27.3 | 48.4 | 20.8 | 1.8 |  | Mar. | | - | 1.2 | 30.2 | | 40.8 | 24.9 | 2.9 | |
| Apr. | - | 0.6 | 17.3 | 48.8 | 27.6 | 5.8 |  | Apr. | - | - | 21.5 | 46.4 | 26.4 | 5.8 |  | Apr. | | - | 0.3 | 17.3 | | 45.8 | 29.1 | 7.6 | |
| May | - | 0.3 | 17.3 | 39.6 | 32.0 | 10.9 |  | May | - | - | 16.7 | 39.6 | 33.4 | 10.3 |  | May | | - | 1.2 | 16.7 | | 39.3 | 30.2 | 12.6 | |
| Jun. | - | - | 14.0 | 39.0 | 31.7 | 15.2 |  | Jun. | - | - | 15.9 | 39.8 | 29.7 | 14.7 |  | Jun. | | - | - | 15.3 | | 34.6 | 34.9 | 15.3 | |
| Jul. | - | - | 12.1 | 35.9 | 36.8 | 15.3 |  | Jul. | - | - | 11.2 | 35.6 | 40.6 | 12.6 |  | Jul. | | - | - | 10.3 | | 38.1 | 38.1 | 13.5 | |
| Aug. | - | - | 12.0 | 32.3 | 36.4 | 19.4 |  | Aug. | - | - | 10.6 | 34.6 | 34.9 | 19.9 |  | Aug. | | - | - | 7.1 | | 35.6 | 37.9 | 19.4 | |
| Sep. | - | - | 9.7 | 33.9 | 38.5 | 17.9 |  | Sep. | - | - | 11.2 | 33.6 | 39.1 | 16.1 |  | Sep. | | - | - | 11.5 | | 32.4 | 34.5 | 21.5 | |
| Oct. | - | 0.6 | 16.9 | 40.2 | 31.7 | 10.7 |  | Oct. | - | - | 13.6 | 40.7 | 36.5 | 9.2 |  | Oct. | | - | - | 16.0 | | 42.1 | 32.6 | 9.2 | |
| Nov. | - | 1.2 | 19.3 | 46.0 | 28.2 | 5.2 |  | Nov. | - | 0.6 | 21.4 | 46.2 | 29.7 | 2.1 |  | Nov. | | - | - | 22.3 | | 45.9 | 27.5 | 4.3 | |
| Dec. | - | 1.5 | 31.0 | 46.4 | 19.0 | 2.1 |  | Dec. | - | 1.2 | 27.4 | 48.2 | 20.2 | 3.0 |  | Dec. | | - | - | 30.4 | | 51.2 | 17.0 | 1.5 | |
| Year | - | 0.9 | 20.7 | 41.7 | 27.8 | 9.0 |  | Year | - | 0.6 | 20.2 | 42.5 | 28.5 | 8.2 |  | Year | | - | 0.6 | 20.2 | | 41.9 | 28.0 | 9.4 | |
| 09:00 UTC | | | | | | |  | 12:00 UTC | | | | | | |  | 15:00 UTC | | | | | | | | | |
| Month | Thermal stress categories | | | | | |  | Month | Thermal stress categories | | | | | |  | Month | | Thermal stress categories | | | | | | | |
|  | 0 | -1 | -2 | -3 | -4 | -5 |  |  | 0 | -1 | -2 | -3 | -4 | -5 |  |  |  | 0 | -1 | -2 | | -3 | -4 | -5 | |
| Jan. | 0.6 | 9.1 | 38.6 | 42.2 | 8.4 | 1.0 |  | Jan. | - | 14.6 | 38.3 | 39.6 | 6.5 | 1.0 |  | Jan. | | 0.6 | 13.6 | 44.5 | | 35.1 | 5.8 | 0.3 | |
| Feb. | 0.3 | 3.5 | 31.9 | 49.7 | 13.5 | 1.0 |  | Feb. | 0.7 | 10.1 | 41.7 | 36.1 | 10.4 | 1.0 |  | Feb. | | 1.4 | 10.4 | 38.2 | | 40.3 | 9.0 | 0.7 | |
| Mar. | - | 2.1 | 29.0 | 45.7 | 20.8 | 2.3 |  | Mar. | 0.3 | 6.2 | 30.2 | 47.8 | 12.9 | 2.6 |  | Mar. | | - | 9.7 | 34.6 | | 40.5 | 12.9 | 2.3 | |
| Apr. | - | - | 17.3 | 47.0 | 28.8 | 7.0 |  | Apr. | - | 0.6 | 20.6 | 43.9 | 27.9 | 7.0 |  | Apr. | | - | 5.2 | 23.0 | | 43.6 | 22.7 | 5.5 | |
| May | - | - | 14.7 | 41.3 | 34.3 | 9.7 |  | May | - | 0.3 | 15.6 | 41.0 | 32.4 | 10.6 |  | May | | - | 1.5 | 19.2 | | 41.9 | 28.3 | 9.1 | |
| Jun. | - | - | 15.9 | 36.1 | 29.7 | 18.3 |  | Jun. | - | 0.3 | 14.7 | 38.0 | 30.7 | 16.3 |  | Jun. | | - | 0.6 | 15.9 | | 37.6 | 31.8 | 14.1 | |
| Jul. | - | - | 9.7 | 37.4 | 37.9 | 15.0 |  | Jul. | - | - | 12.9 | 37.9 | 32.9 | 16.2 |  | Jul. | | - | - | 15.3 | | 38.5 | 31.8 | 14.4 | |
| Aug. | - | - | 10.9 | 34.4 | 32.9 | 21.8 |  | Aug. | - | - | 9.1 | 34.1 | 33.8 | 22.9 |  | Aug. | | - | 1.2 | 17.6 | | 34.0 | 30.8 | 16.4 | |
| Sep. | - | - | 12.7 | 35.5 | 32.1 | 19.7 |  | Sep. | - | 1.8 | 17.6 | 28.8 | 36.1 | 15.8 |  | Sep. | | - | 5.8 | 18.8 | | 33.0 | 29.4 | 13.0 | |
| Oct. | - | - | 16.9 | 36.8 | 37.4 | 8.9 |  | Oct. | 0.3 | 2.7 | 18.4 | 42.1 | 29.7 | 6.8 |  | Oct. | | 0.9 | 5.0 | 24.0 | | 38.9 | 25.2 | 5.9 | |
| Nov. | 0.3 | 4.6 | 23.9 | 44.6 | 22.6 | 4.0 |  | Nov. | 0.6 | 5.5 | 30.0 | 39.8 | 21.1 | 3.1 |  | Nov. | | 0.9 | 6.1 | 35.2 | | 39.8 | 16.2 | 1.8 | |
| Dec. | - | 3.3 | 34.2 | 50.3 | 11.3 | 0.9 |  | Dec. | 0.6 | 7.5 | 37.9 | 40.9 | 11.9 | 1.2 |  | Dec. | | 0.6 | 10.1 | 38.4 | | 41.4 | 9.2 | 0.3 | |
| Year | 0.1 | 1.8 | 21.1 | 41.6 | 26.1 | 9.3 |  | Year | 0.2 | 4.0 | 23.6 | 39.2 | 24.1 | 8.9 |  | Year | | 0.4 | 5.7 | 26.8 | | 38.7 | 21.3 | 7.1 | |
| 18:00 UTC | | | | | | |  | 21:00 UTC | | | | | | |  |  | | | | | | | | | |
| Month | Thermal stress categories | | | | | |  | Month | Thermal stress categories | | | | | |  | | | | | |  |  |  |  |  |
|  | 0 | -1 | -2 | -3 | -4 | -5 |  |  | 0 | -1 | -2 | -3 | -4 | -5 |  | |  | | | | | | | |  |
| Jan. | 1.3 | 10.7 | 49.5 | 31.1 | 6.5 | 1.0 |  | Jan. | - | 8.1 | 43.7 | 40.8 | 6.5 | 1.0 |  | |  |  |  |  |  |  |  |  |  |
| Feb. | 0.7 | 10.5 | 39.4 | 41.5 | 7.0 | 1.0 |  | Feb. | 0.7 | 6.6 | 34.6 | 46.0 | 10.7 | 1.4 |  | |  |  |  |  |  |  |  |  |  |
| Mar. | 0.6 | 7.6 | 34.9 | 42.2 | 12.9 | 1.8 |  | Mar. | - | 3.2 | 29.6 | 44.6 | 19.4 | 3.2 |  | |  |  |  |  |  |  |  |  |  |
| Apr. | - | 3.9 | 21.5 | 47.0 | 21.5 | 6.1 |  | Apr. | - | 0.9 | 17.9 | 47.6 | 27.3 | 6.4 |  | |  |  |  |  |  |  |  |  |  |
| May | - | 1.2 | 18.5 | 42.5 | 27.6 | 10.3 |  | May | - | 0.6 | 16.1 | 35.8 | 36.7 | 10.9 |  | |  |  |  |  |  |  |  |  |  |
| Jun. | - | - | 15.9 | 35.2 | 32.7 | 16.2 |  | Jun. | - | - | 13.4 | 40.9 | 31.1 | 14.6 |  | |  |  |  |  |  |  |  |  |  |
| Jul. | - | - | 11.2 | 39.1 | 33.8 | 15.9 |  | Jul. | - | - | 11.8 | 36.5 | 36.5 | 15.3 |  | |  |  |  |  |  |  |  |  |  |
| Aug. | - | 1.8 | 19.1 | 32.3 | 30.8 | 16.1 |  | Aug. | - | - | 10.3 | 32.4 | 37.4 | 20.3 |  | |  |  |  |  |  |  |  |  |  |
| Sep. | - | 3.9 | 18.2 | 36.4 | 27.9 | 13.6 |  | Sep. | - | - | 11.2 | 34.8 | 34.2 | 19.7 |  | |  |  |  |  |  |  |  |  |  |
| Oct. | 0.3 | 5.7 | 23.5 | 38.4 | 27.1 | 5.1 |  | Oct. | - | 2.4 | 15.8 | 42.6 | 31.8 | 7.4 |  | |  |  |  |  |  |  |  |  |  |
| Nov. | 0.3 | 4.6 | 34.0 | 42.6 | 17.2 | 1.2 |  | Nov. | - | 5.5 | 22.4 | 46.6 | 23.3 | 2.1 |  | |  |  |  |  |  |  |  |  |  |
| Dec. | 1.2 | 11.9 | 36.6 | 40.5 | 8.9 | 0.9 |  | Dec. | 0.0 | 5.7 | 35.4 | 46.1 | 11.3 | 1.5 |  | |  |  |  |  |  |  |  |  |  |
| Year | 0.4 | 5.0 | 26.5 | 39.1 | 21.4 | 7.6 |  | Year | 0.1 | 2.7 | 21.6 | 41.1 | 25.8 | 8.8 |  | |  |  |  |  |  |  |  |  |  |

**Table S3** Frequency [%] of occurrence of single hours (a) and their sequences lasting from 2 to 6 hours (b) during the day in individual months without thermal stress (with thermoneutral conditions – 0) or slight cold stress (-1) at Arctowski Station (2013-2023)

a) single hours

| months/hours | 0 | 1 | 2 | 3 | 4 | 5 | 6 | 7 | 8 | 9 | 10 | 11 | 12 | 13 | 14 | 15 | 16 | 17 | 18 | 19 | 20 | 21 | 22 | 23 | sum |
| --- | --- | --- | --- | --- | --- | --- | --- | --- | --- | --- | --- | --- | --- | --- | --- | --- | --- | --- | --- | --- | --- | --- | --- | --- | --- |
| Jan. | 0.0 | 0.1 | 0.1 | 0.2 | 0.3 | 0.4 | 0.4 | 0.5 | 0.6 | 0.7 | 0.7 | 0.8 | 0.9 | 0.9 | 1.0 | 1.1 | 1.2 | 1.2 | 1.3 | 1.4 | 1.5 | 1.5 | 1.6 | 1.7 | 20.1 |
| Feb. | 0.0 | 0.1 | 0.1 | 0.1 | 0.4 | 0.1 | 0.1 | - | 0.2 | 0.3 | 0.4 | 0.4 | 0.9 | 0.7 | 0.1 | 0.7 | 0.5 | 0.6 | 0.5 | 0.2 | 0.4 | 0.3 | 0.4 | 0.1 | 7.5 |
| Mar. | 0.1 | 0.0 | - | 0.1 | 0.1 | 0.1 | - | 0.0 | - | 0.2 | 0.3 | 0.4 | 0.4 | 0.4 | 0.5 | 0.4 | 0.1 | 0.4 | 0.3 | 0.4 | 0.2 | 0.1 | 0.0 | 0.1 | 4.5 |
| Apr. | 0.1 | 0.1 | 0.0 | - | 0.1 | 0.1 | 0.1 | 0.1 | - | - | 0.0 | 0.4 | 0.4 | 0.2 | 0.5 | 0.4 | 0.4 | 0.3 | 0.4 | 0.4 | 0.1 | 0.0 | 0.1 | 0.0 | 3.9 |
| May | 0.0 | 0.1 | - | - | 0.1 | 0.0 | 0.0 | 0.0 | 0.0 | - | - | 0.0 | - | - | 0.2 | 0.4 | 0.1 | 0.1 | 0.1 | 0.0 | 0.0 | 0.0 | - | 0.0 | 1.2 |
| Jun. | - | - | - | - | - | - | - | - | 0.0 | - | - | - | 0.0 | - | - | 0.0 | - | 0.0 | - | - | - | - | 0.0 | - | 0.0 |
| Jul. | - | - | - | - | - | - | - | - | - | - | - | - | - | - | - | - | 0.0 | 0.0 | - | - | - | - | - | - | 0.0 |
| Aug. | - | - | - | - | - | - | - | - | - | - | - | - | - | 0.0 | 0.0 | 0.0 | 0.0 | 0.0 | 0.0 | 0.0 | - | - | - | - | 0.0 |
| Sep. | - | - | - | - | - | - | - | - | 0.0 | - | - | - | 0.0 | 0.0 | 0.1 | 0.0 | 0.1 | 0.1 | 0.0 | 0.0 | 0.0 | - | - | - | 0.3 |
| Oct. | 0.0 | - | - | - | - | - | - | - | - | - | 0.0 | 0.1 | 0.2 | 0.2 | 0.1 | 0.4 | 0.2 | 0.1 | 0.0 | 0.1 | 0.0 | 0.0 | 0.0 | - | 1.4 |
| Nov. | 0.1 | 0.0 | 0.0 | 0.0 | 0.0 | 0.0 | - | 0.0 | 0.0 | 0.0 | 0.1 | 0.1 | 0.1 | 0.1 | 0.3 | 0.3 | 0.3 | 0.2 | 0.1 | 0.3 | 0.1 | 0.0 | 0.0 | 0.0 | 2.1 |
| Dec. | 0.0 | 0.1 | 0.0 | 0.0 | 0.0 | 0.0 | - | 0.1 | 0.0 | 0.4 | 0.4 | 0.1 | 0.1 | 0.5 | 0.1 | 0.5 | 0.5 | 0.1 | 0.0 | 0.1 | 0.3 | 0.2 | 0.1 | 0.0 | 3.6 |
| Year | 0.2 | 0.4 | 0.3 | 0.4 | 0.9 | 0.7 | 0.7 | 0.7 | 0.8 | 1.5 | 1.8 | 2.3 | 2.9 | 3.1 | 2.9 | 4.2 | 3.4 | 3.2 | 2.8 | 2.8 | 2.5 | 2.1 | 2.1 | 1.9 | 100.0 |

b) 2-6 hours sequences

| months/hours | 0 | 1 | 2 | 3 | 4 | 5 | 6 | 7 | 8 | 9 | 10 | 11 | 12 | 13 | 14 | 15 | 16 | 17 | 18 | 19 | 20 | 21 | 22 | 23 | sum |
| --- | --- | --- | --- | --- | --- | --- | --- | --- | --- | --- | --- | --- | --- | --- | --- | --- | --- | --- | --- | --- | --- | --- | --- | --- | --- |
| Jan. | - | 0.2 | 0.5 | 0.2 | 1.1 | 0.2 | 0.2 | 0.7 | 0.7 | 0.9 | 1.1 | 1.4 | 2.7 | 2.1 | 0.5 | 2.3 | 1.6 | 1.8 | 1.6 | 0.7 | 1.1 | 0.9 | 1.1 | 0.5 | 24.3 |
| Feb. | 0.2 | - | 0.2 | 0.2 | 0.2 | 0.5 | 0.2 | - | 0.2 | 0.7 | 0.9 | 1.4 | 1.1 | 1.1 | 1.6 | 1.1 | 0.5 | 1.4 | 0.9 | 1.1 | 0.7 | 0.2 | - | 0.2 | 14.9 |
| Mar. | 0.2 | 0.2 | - | 0.5 | 0.5 | 0.2 | 0.5 | 0.2 | - | 0.5 | - | 1.1 | 1.1 | 0.7 | 1.6 | 1.1 | 1.1 | 0.9 | 1.1 | 1.1 | 0.2 | - | 0.2 | - | 13.3 |
| Apr. | - | 0.2 | - | - | 0.2 | - | - | - | - | - | - | - | 0.2 | 0.2 | 0.7 | 1.4 | 0.5 | 0.2 | 0.5 | - | - | - | - | - | 4.1 |
| May | - | - | - | - | - | - | - | 0.2 | - | - | 0.2 | 0.2 | - | - | - | - | 0.2 | - | 0.2 | - | - | - | - | - | 1.1 |
| Jun. | - | - | - | - | - | - | - | 0.2 | - | - | - | - | - | - | - | - | - | - | - | - | - | - | - | - | 0.2 |
| Jul. | - | - | - | - | - | - | - | - | - | - | - | - | - | - | - | - | - | - | - | - | - | - | - | - | 0.0 |
| Aug. | - | - | - | - | - | - | - | - | - | - | - | - | - | - | 0.2 | - | 0.2 | 0.5 | - | - | - | - | - | - | 0.9 |
| Sep. | - | - | - | - | - | - | - | - | - | - | - | 0.2 | 0.7 | 0.7 | 0.2 | 1.4 | 0.7 | 0.2 | - | 0.2 | - | - | - | - | 4.3 |
| Oct. | 0.2 | - | - | - | - | - | - | - | - | - | 0.2 | 0.2 | 0.2 | 0.5 | 0.9 | 0.9 | 0.9 | 0.7 | 0.5 | 0.9 | 0.5 | - | - | - | 6.6 |
| Nov. | - | 0.5 | - | - | - | - | - | 0.2 | - | 1.1 | 1.1 | 0.5 | 0.5 | 1.6 | 0.2 | 1.6 | 1.6 | 0.5 | - | 0.2 | 0.9 | 0.7 | 0.2 | - | 11.4 |
| Dec. | 0.2 | - | - | - | - | - | - | 0.5 | 0.9 | 0.5 | - | 0.2 | 2.1 | 2.3 | 1.8 | 1.8 | 1.4 | 2.3 | 1.6 | 1.4 | 0.9 | 0.2 | 0.2 | 0.5 | 18.8 |
| Year | 0.9 | 1.1 | 0.7 | 0.9 | 2.1 | 0.9 | 0.9 | 2.1 | 1.8 | 3.7 | 3.7 | 5.3 | 8.7 | 9.2 | 7.8 | 11.7 | 8.7 | 8.5 | 6.4 | 5.7 | 4.3 | 2.1 | 1.8 | 1.1 | 100.0 |
